# Arthrofibrosis is a common but poorly defined complication in multiligament knee injuries: a systematic review

**DOI:** 10.1007/s00402-022-04730-9

**Published:** 2022-12-15

**Authors:** Hendrik Fahlbusch, Lukas Krivec, Sebastian Müller, Alonja Reiter, Karl Heinz Frosch, Matthias Krause

**Affiliations:** 1 Department of Trauma and Orthopaedic Surgery, University Medical Center Hamburg-Eppendorf, Hamburg, Germany; 2 Department of Orthopaedics, University Hospital Basel, Basel, Switzerland; 3 Department of Trauma Surgery, Orthopaedics and Sports Traumatology, BG Hospital Hamburg, Hamburg, Germany

**Keywords:** Knee, Multiligament knee injury, Knee dislocation, Arthrofibrosis, Stiffness, Systematic review

## Abstract

**Purpose:**

The purpose of this study is to systematically review multiligament knee injury (MLKI) outcome studies to determine definitions of arthrofibrosis (AF) and provide information about incidence, management as well as potential risk factors.

**Methods:**

A systematic literature search was performed (PubMed and Cochrane library) following the PRISMA guidelines of operatively treated MLKI (Schenck II–IV) studies reporting the incidence of AF. Twenty-five studies met the inclusion criteria. Injury pattern, timing of surgery, surgical technique, treatment of AF, rehabilitation programs and PROMS were inquired. Risk of bias and quality of evidence were assessed using the Coleman methodological score.

**Results:**

Twenty-five studies with a total of 709 patients with a mean age of 33.6 ± 4.8 years were included and followed 47.2 ± 32.0 months. The majority of studies (22/25) used imprecise and subjective definitions of AF. A total of 86 patients were treated for AF, resulting in an overall prevalence of 12.1% (range 2.8–57.1). Higher-grade injuries (Schenck III–IV), acute treatment and ROM (range of motion) limiting rehabilitation programs were potential risk factors for AF. The time from index surgery to manipulation anesthesia (MUA) and arthroscopic lysis of adhesions (LOA) averaged at 14.3 ± 8.8 and 27.7 ± 12.8 weeks. Prior to MUA and LOA, the ROM was 51.7° ± 23.5 and 80.2° ± 17.0, resulting in a total ROM gain after intervention of 65.0° ± 19.7 and 48.0° ± 10.6, respectively; with no reports of any complication within the follow-up. The overall methodological quality of the studies was poor as measured by the Coleman score with average 56.3 ± 12.5 (range 31–84) points.

**Conclusions:**

AF is a common but poorly defined complication particularly in high-grade MLKI. Early postoperative and intensified physiotherapy is important to reduce the risk of AF. MUA and LOA are very effective treatment options and result in good clinical outcome. Prospective studies with bigger study population are needed to optimize treatment algorithms of further patients after MLKI.

The protocol of this systematic review has been prospectively registered with PROSPERO (CRD42021229187, January 4th, 2021).

## Introduction

Multiple-ligament injuries of the knee (MLKI) are rare (0.02–0.2% of all orthopedic injuries), but often devastating in nature as they are potentially limb-threatening, given the possibility of concomitant popliteal artery injuries with amputations being described in up to 25.0% [6, 9, 26, 45].

Historically, these injuries have been managed conservatively in the acute phase due to the reported high risk of arthrofibrosis (AF) [12, 23]. In the last decades, there has been a strong trend toward surgical management of such injuries, since recent studies provided evidence of worse functional outcomes, persisting instability and contracture in patients managed non-operatively [50, 67, 71]. However, there are many controversies with regard to type and timing of the surgical management [7, 60]. Strategies range from early to late surgery and repair to reconstruction and one- to two-stage procedures [7].

Commonly reported complications after MLKI surgery include wound infection, deep venous thrombosis and AF [23, 53], with MLKI having a much greater risk of complication compared to single ligament injuries [5, 9, 16, 71]. The overall complication rate ranges from 6.0% to 75.0% and is directly linked to number of injured ligaments [3, 9, 13, 86]. With rates up to 57.0%, AF is the most common complication and often requires interventional treatment [7, 43, 59, 64, 72, 76]. It has long been recognized but definitions widely vary and treatment guidelines are still lacking. Histopathologically, AF is caused by prolonged expression of inflammatory cytokines, migration of myofibroblasts, resulting in increased scar tissue and eventually leading to clinically apparent loss of motion of the knee [11, 82]. This extensive scar tissue is used in the concept of ligament bracing in a sense of “guided arthrofibrosis” for treating acute knee dislocations [25].

In addition to extensive injuries and complicated surgeries [59], several factors are assumed to increase the risk of stiffness such as injury of two or more ligaments, repair of medial sided structures and acute surgery within 3 weeks [9, 13, 41]. Protection of repaired or reconstructed structures, e.g., by means of bracing, restrictions of weight bearing and/or range of motion are still considered important to enable healing of these structures. Likewise, a good balance between protective measures and early enforced rehabilitation, assuming to potentially decrease the likelihood of AF, is of crucial importance [52]. Several protocols have already published recommendations, aiming to minimize the risk of AF such as staged, delayed surgery or the use of a hinged external fixator [2, 5, 49, 84]. However, to date, there is still no consensus on the best surgical procedure and rehabilitation program following MLKI surgery to avoid AF.

The purpose of this study is to systematically review MLKI outcome studies to determine definitions of AF and provide information about incidence, management and potential risk factors of this complication. We hypothesized that AF is inconsistently defined and more severely MLKI and acute surgery result in higher rates of AF. Furthermore, the treatment of AF is mainly operative and shows good results.

## Materials and methods

### Search criteria

A search for relevant studies that met prespecified inclusion criteria was conducted by two independent reviewers (H.F. and L.K.) on August 31st, 2021, through the two major electronic databases PubMed and Cochrane library.

The search strategy included the two following keyword searches:

1. "knee dislocation" OR "multiligament* knee injury*" OR "Tibiofemoral dislocation" OR "multiligament knee reconstruction" OR "multiligament-injured knee."
2. ("anterior cruciate ligament" OR acl OR pcl OR "posterior cruciate ligament") AND ("stiffness" OR "range of motion deficits" OR "ROM deficits" OR "arthrofibrosis") AND ("reconstruction" OR "treatment" OR "surgery" OR "repair").

Both keyword searches were merged carefully afterward. Search terms, if possible, were mapped to relevant MeSH terms and subject headings. A supplementary search of the reference list of relevant articles was also conducted. The study was performed as a systematic review of the current literature following the PRISMA (Preferred Reporting Items for Systematic Reviews and Meta-analyses) guidelines [58]. The protocol of this systematic review has been prospectively registered with PROSPERO (CRD42021229187, January 4th, 2021).

### Study selection

In a first step, title and abstract of each study were evaluated to meet inclusion criteria (see Table 1).

**Table 1:** Study Inclusion and Exclusion Criteria

Inclusion criteria	Exclusion criteria
Report of AF or stiffness following MLKI requiring interventional treatment like lysis of adhesions (LOA) or manipulation under anesthesia (MUA)	Articles that have investigated the outcome of conservatively treated injuries or studies older than 1990s
MLKI defined as the disruption of at least both cruciate ligaments (Schenck grade II–IV)	Reports on guidelines, technique articles, reviews or systematic reviews
Adult female and male patients	Complex fracture dislocation, such as tibial fracture and/or distal femur fracture requiring open reduction and internal fixation
Full texts available in English or German language	

Schenck I injuries are very common and differentiate from higher-grade injuries in terms of trauma mechanisms, treatment and outcome. On the other hand, Schenck V injuries are rare but often need complex internal fixation and are rather heterogeneous among themselves, since the description of fracture-dislocation injuries is often limited. As non-operative therapy is reserved for individual rare cases [15, 18, 22, 38, 67, 83] and data of MLKI are very inhomogeneous, this review focuses particularly on operatively treated knee dislocations with at least two torn cruciate ligaments without reported fracture dislocations.

Noticeably, studies including Schenck I or V injuries, where an individual analysis of Schenck II–IV injuries was possible, were also included. In cases where the review of the title and abstract did not clearly indicate whether a study was suitable for inclusion, the full-text article was analyzed. Two reviewers (H.F. and L.K.) independently evaluated the selected articles for meeting the inclusion criteria. The decision to include or exclude the study was made based on a group consensus. Any deviations from consensus were discussed and resolved as a group. Additionally, all references from the included studies were reviewed and reconciled to verify that no relevant articles were missing.

### Data extraction

Two independent reviewers (H.F. and L.K.) performed data extraction in duplicates and used a form specifically designed for this review. The following data were extracted from each study: Number of participants included average age of participants, time of follow-up, injury pattern (Schenck Classification), surgical technique, timing of surgery, postoperative rehabilitation program and PROMS (Lysholm and IKDC Score). Furthermore, rate of AF, distribution and timing of LOA/MUA, range of motion (ROM) before and ROM gain after LOA/MUA were considered in the analysis.

### Study quality assessment

The rating of evidence level based on Wright et al. [88] was used for this review. The quality of the studies was assessed using the Coleman methodological score [8], which was developed to assess the quality of primary studies in terms of risk of bias and applicability concerns. The total score can range from 0 to 100, and higher scores are indicative of absence of bias and confounding factors. The final score was categorized as excellent (85–100 points), good (70–84 points), fair (50–69 points) and poor (< 50 points) [56]. Each included study was assigned a score independently by the two reviewers (H.F. and L.K). Disagreements between the evaluators were resolved by consensus, with a third evaluator (M.K.) being called in, when consensus could not be reached.

### Statistical analysis

Statistical analysis was performed using SPSS 15.0 software (SPSS Inc., Chicago, IL). Because of limitations in reporting (lack of availability AF/Stiffness) as well as heterogeneity between studies, a meta-analysis was not be performed. For descriptive purposes, the rate of AF (weighting based on the study sample size), averages for time to LOA/MUA, total ROM before and total ROM gain after LOA/MUA were calculated from the study summary data. The interrater reliabilities were obtained to assess the agreement among the two observers (H.F. and L.K.) for determining the Coleman score. Cohen’s kappa was evaluated between the average scores. A *p* value of less than 0.05 was considered significant.

## Results

### Literature selection

**Fig. 1:**
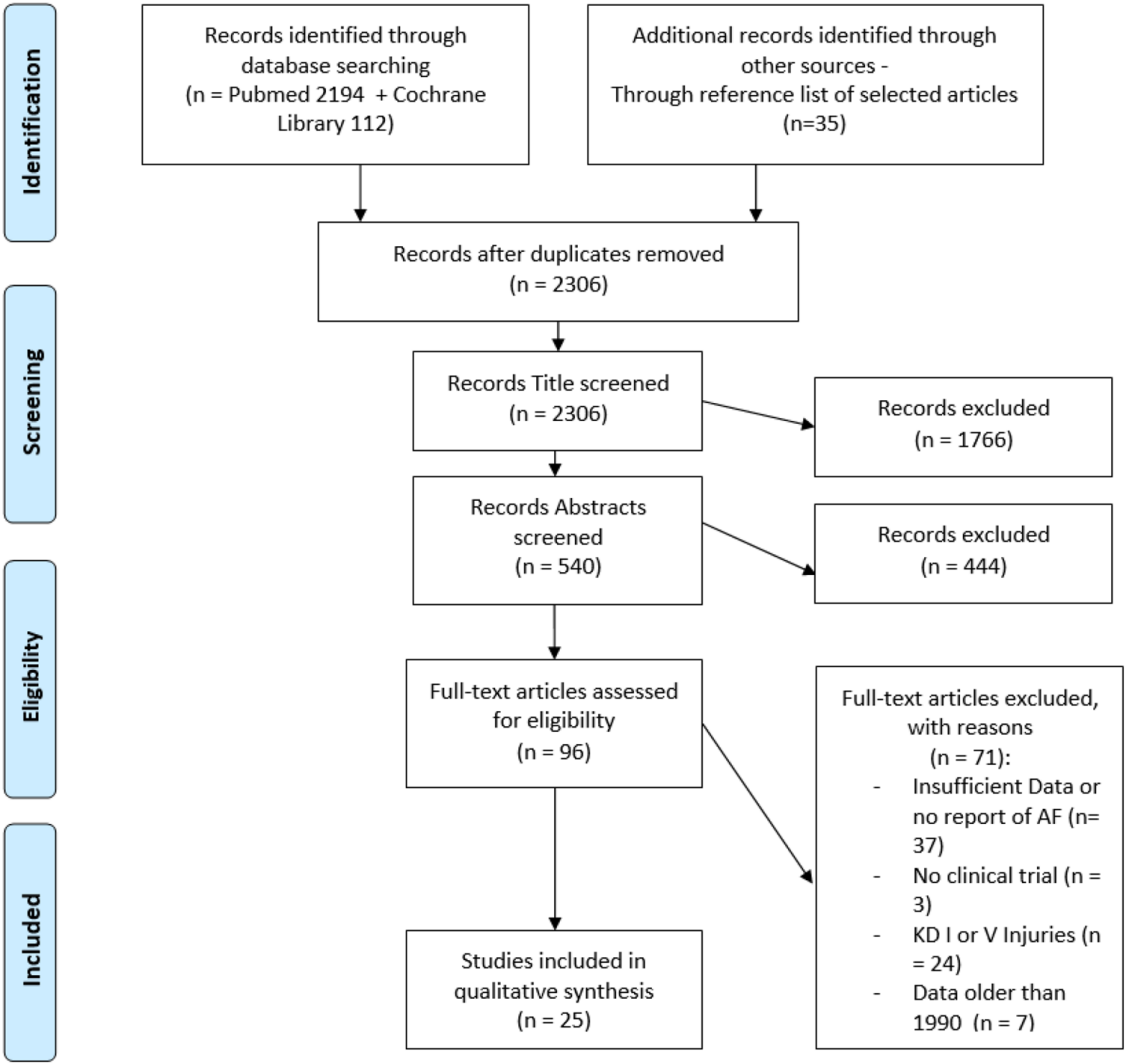
Preferred Reporting Items for Systematic Reviews (PRISMA) flowchart demonstrating the article selection process

A total of 2341 articles were identified, after removal of duplicates (*n* = 35), 2,06 titles and abstracts were screened for eligibility (see Fig. 1). After exclusion of 2210 articles through screening, 96 remaining articles underwent a full-text analysis by the reviewers to evaluate matching of inclusion and exclusion criteria (see Table 1). Any discrepancies were mutually resolved. Ultimately, 25 articles were included, assessed and underwent a quality review.

### Study characteristics

In total, the studies included 709 patients after surgical treatment of MLKI, of whom 12.1% (*n* = 86) were diagnosed with postoperative AF. The average age of included patients was 33.6 ± 4.8 years with a mean follow-up of 47.2 ± 32.0 months. According to the classification by Schenck, there were 8.5% (*n* = 55) grade II, 44.0% (*n* = 285) grade IIIM, 31.4% (*n* = 203) grade IIIL and 16.1% (*n* = 104) grade IV injuries (*n* = 62 undefined Schenck II–IV injuries).

### Definition

Overall, 22 of 25 studies used subjective definitions that can be divided into three subgroups: (1) Eight studies described AF solely as requirement of interventions like MUA/LOA. (2) Six studies referred to AF as “stiffness” and (3) eight studies referred to different manifestations of limited motion (flexion/extension loss).

Talbot, Richter and Jokela et al. [41, 70, 81] used objective cutoffs and defined AF as < 90° of flexion after 4 weeks; > 20° flexion loss to the contralateral side; knee extension deficiency of more than 10 degrees and flexion deficiency more than 20 degrees, respectively. To achieve simplification and comparability in this review, we defined AF, if not explicitly defined otherwise by the author, as requirement of additional manipulative treatments like LOA and MUA Table 2.

**Table 2:** Characteristics of included studies (*n* = 25)

Author	Year	Type of study	Level of evidence	Mean age (range)	Patients	Mean follow-up (mo)	Schenck classification	Surgical technique	Time to surgery (acute/staged/chronic)	Lysholm (range)	IKDC	Coleman score
Shapiro et al. [76]	1995	Retrospective case series	IV	26.6	7	51.4	II–IV	ACL/PCL reconstruction with allografts; collaterals repair	Acute: 9.6 days (5–14)	74.7 (34–93)	n/a	31
Wascher et al. [84]	1999	Retrospective case series	IV	27.5 (14–51)	13	38.4 (24–54)	IIIM: 54% IIIL: 46%	ACL/PCL reconstruction; collaterals repair	69% Acute: 11 days 31% Chronic: 11 months	88.0 (42–100)	B 50.0% C 42.0% D 8.0%	38
Yeh et al. [89]	1999	Retrospective case series	IV	37.8 (16–65)	23	27.2 ± 7.86	IIIM: 57% IIIL: 30% IV: 13%	Reconstruction of PCL (autograft + 1allograft); No ACL repair; Collaterals repair	Acute: 11.5 ± 5 days	84.1 (79–93)	n/a	49
Ohkoshi al. [62]	2002	Retrospective case series	IV	28.7 (18–27)	8	40.1 ±16.7	IIIM: 67% IIIL: 33%	ACL/PCL reconstruction (autograft and artificial ligament); collaterals reconstruction (Autograft)	Staged: 1: 12.8 ± 5.3 days 2. 3.8 ± 1.1 months (after achieving sufficient ROM)	n/a	B 77.8% C 22.2%	43
Harner al. [22]	2004	Retrospective cohort study	III	28.4	31	> 24.0	III	ACL/PCL reconstruction (allograft); collaterals reconstruction (allograft) or repair	Acute: 12 days (5–21) Chronic: 6.5 months (5 weeks–22 months)	87.0 ± 12.7	B 35.0% C 39.0% D 26.0%	54
Talbot et al. [81]	2004	Retrospective case series	IV	28.5 (15–73)	20	27.4	II: 5% IIIM: 43% IIIL: 48% IV: 5%	ACL/PCL repair and augmentation with LARS; collaterals repair and if necessary Augmentation with LARS	Acute: 11 days	71.7 ± 18	n/a	57
Bin et al. [5]	2007	Retrospective case series	IV	30.4 (20–51)	14	88.9 (35–110)	IIIM: 47% IIIL: 33% IV: 20%	ACL/PCL reconstruction with allo/autograft; MCL repair or conservative; LCL repair or reconstruction	Staged: 1: < 2 Weeks to injury 2. After full ROM (3-6monts)	87.6	A 20.0% B 53.0% C 27.0%	50
Ibrahim et al. [34]	2008	Retrospective case series	IV	27.3 (17–45)	20	53.0 (36–96)	IIIM: 75% IIIL: 25%	ACL/PCL reconstruction (autograft); collaterals reconstruction with LARS	Acute: 2–3 weeks	97–80: 95% 78–79: 5%	B 45.0% C 45.0% D 10.0%	54
Lo et al. [51]	2009	Prospective clinical trail	III	33 (19–48)	11	55.0 (36–78)	II–III	ACL/PCL reconstruction (autograft); MCL repair; PLC reconstruction (autograft)	Chronic: 76 days (30–150)	88.0 ± 5.8	A + B 82.0%	70
Engebretsen et al. [13]	2009	Prospective cohort study	III	33 (12–82)	85	Minimum 24.0 (25.2–118.8)	II: 6% IIIM: 49% IIIL: 32% IV: 10 12%	ACL/PCL reconstruction (auto-/allograft, collaterals repair or reconstruction	Acute 60%: 1–2 weeks Chronic 40%: > 2 weeks	81.0 (42–100)	64.0 ± 20.0	83
Ranger et al. [69]	2011	Retrospective case series	IV	38.5	71	24–96	II: 4% IIIL: 39% IIIM: 41% IV: 16%	All structures repair and reconstruction with LARS (ligament augmentation and reconstruction system)	Acute: 10.8 days (± 8)	78.5 ± 18.5	67.9 ± 19.9	55
Ibrahim et al. [35]	2013	Retrospective case series	IV	26.4 (18–48)	20	44.0 (24–52)	IIIL	ACL/PCL reconstruction (autograft/LARS); PLC reconstruction (autograft)	Acute: 15–21 days	95–80 90% 75–79 10%	B 45.0% C 45.0% D 10.0%	49
Piontek et al. [68]	2013	Retrospective cohort study	III	36.0	11	27.0 ± 4	II: 64% IIIM: 36%	All structures reconstruction (autograft)	Chronic: > 6 months	100–98 54.6% 97–93 45.4%	A 36.4% B 54.6% C 9.0%	66
Werner et al. [87]	2014	Retrospective case series	IV	35.0	65	144.0	IIIM: 49% IV: 51%	ACL reconstruction; PCL reconstruction in 63%; medial: 25% conservative, 18% repair and 57% reconstruction	Acute: < 3 weeks	KD IIIM: 88.0 KD IV: 67.0	n/a	60
Heitmannet al. [25]	2014	Prospective case series	III	33.0 (18–60)	8	11.8 (10–15)	III–IV	ACL/PCL repair and bracing; MCL repair	Acute: 5 days (4–7)	85.3 (62–99)	75.7 (52.9–94.3)	65
Richter et al. [70]	2014	Retrospective case series	IV	28.0 (16–39)	8	72 (24–134.4)	IIIM	ACL/PCL reconstruction (allograft); MCL reconstruction (allograft)	25% acute 75% chronic	81.0 (58–100)	B 57.0% C 29.0% D 14.0%	52
Kohl et al. [46]	2015	Prospective case series	III	33.4 (17–56)	35	25.9 (12–42)	III: 74.3% IV: 25.7%	ACL repair and augmentation (dynamic intraligamentary stabilization); PCL repair; collaterals repair or combined reconstruction (autograft)	Acute: < 2 days	90.8 (81–95)	B 83.0% C 17.0%	63
Angelini et al. [1]	2015	Retrospective case series	IV	29.3	14	49.4	IIIM 14% IIIL 64% IV 22%	All ligaments reconstruction (allografts); hinged external fixateur for 6 weeks postoperative	Chronic: 2.5 months (0.5–3)	81.5 (49–95)	B 71.0% C 21.0% D 8.0%	48
Khakha et al. [43]	2016	Retrospective cohort study	III	36.5 (19–65)	36	121.2 (84–228)	II–IV	ACL/PCL reconstruction (auto-/allograft) with LARS; collaterals reconstruction or repair	Acute: 12 days (1–21)	80.0 (57–91)	A 3.0% B 56.0% C 36.0% D 8.0%	55
Huax et al. [30]	2016	Retrospective case series	IV	38.8	16	57.6 ± 15.6	IIIM 17% IIIL 22% IV 50% V 11%	All ligaments repair	Acute: 5–10 days	87.5 ± 7.7	n/a	60
Sundararajan et al. [80]	2018	Retrospective cohort study	IV	39.0 (17–74)	45	36.0 (24–72)	IIIM 69% IIIL 31%	ACL/PCL reconstruction (autograft); collaterals repair or reconstruction; MCL mostly conservative	78% acute-subacute: < 6 weeks 11% subacute: (6–12 weeks) 11% chronic: (3 months-6 months)	87.7	74.7	58
Heitmann et al. [24]	2019	Prospective multicenter study	II	34.2 (18–60)	69	14.0	IIIM 35% IIIL 54% IV 12%	ACL/PCL repair and bracing; collaterals repair	Acute: 7.3 ± 1.6 days	81.0 ± 15.5	A 13.0% B 19.0% C 32.0% D 13.0%	84
Jokela et al. [41]	2020	Retrospective cohort study	III	prox: 39.0 (21–64) distal 49.0 (17–67)	25	Prox/mid: 98 (40–145) distal: 66 (24–82)	IIIM	ACL/PCL reconstruction; MCL (proximal/midsubstance) conservative; MCL (distal) repair/reconstruction	Acute: 19 days (5–38)	prox/mid: 88 (57–99) distal: 75 (40–100)	prox/mid: 80.0 (57–99) distal: 62.0 (39–87)	62
Rosteius et al. [75]	2021	Retrospective Cohort Study	III	38.3	27	18.1 ± 12.1	IIIM 22% IIIL 33% IV 45%	All ligaments repair and bracing	Acute	81.5 ± 10.4	n/a	63
Goyal et al. [19]	2021	Retrospective case series	IV	33.5	27	24.0	II 30% IIIM 41% IIIL 22% IV 7%	All ligaments reconstruction (autograft)	Chronic: 14.6 ± 5.9 weeks	50.4 ± 11.7	62.8 ± 5.1	66

Age, follow-up, time to surgery and subjective scores (Lysholm, IKDC) are stated in mean ± standard deviation (range). Results of PROMS (Lysholm and IKDC Score) are stated in points (0–100) or are qualified as “normal” (A), “nearly normal” (B), “abnormal” (C) or “severely abnormal” (D). Acute surgery is defined as surgery within < 3 weeks after trauma

*ACL* Anterior cruciate ligament, *PCL* posterior cruciate ligament, *MCL* medial collateral ligament, *LCL* lateral collateral ligament, *PLC* posterolateral corner, *prox* proximal, *athro* arthroscopically assisted

### Prevalence

In total there were 86 cases of AF in 709 knees, which was equivalent to 12.1% (Table 3). The highest rate of AF was described by Shapiro et al. (57.1%) [76] and lowest by Khak et al. (2.8%) [43]. In both studies, MLKI were treated acutely (< 3 weeks) and reconstruction of both cruciate ligaments with concomitant repair or reconstruction of collateral ligaments was performed.

**Table 3:** Summary of AF cases from included studies divided into subgroups

Parameter	Total	Acute treatment	Chronic treatment [1, 19, 51, 68]	Mixed acute and chronic treatment [70, 80, 84]	Staged treatment [5, 62]	ACL/PCL repair [24, 25, 30, 46, 69, 75, 81]	ACL/PCL reconstruction	ROM week 1–3 allowed ≥ 90°	ROM week 1–3 allowed < 90°[5, 13, 25, 30, 35, 76, 84, 89]
Number of studies	25	16	4	3	2	7	18	11	8
Total number of patients	709	558	63	66	22	246	463	267	186
AF in % (*n*)	12.1 (86)	12.7 (71)	9.5 (6)	7.6 (5)	18.2 (4)	14.2 (35)	11.0 (51)	11.6 (31)	14.0 (26)

Subgroups include timing of surgery (acute, chronic and staged), surgical technique (repair or reconstruction of cruciate ligaments) and early postoperative rehabilitation (ROM restriction greater or under 90° Flexion at week 0–2 postoperatively)

### Risk factors

#### Injury pattern

There was a trend toward higher rates of AF in Schenck III and IV injuries (Table 4). This was especially supported by Huax et al. [30], where all knees with AF had Schenck IV injuries. Also, Schenck II injuries [22] tended to have lower rates compared to Schenck III–IV injuries. On the other hand, Axibal et al. [3] could not find an increased rate comparing Schenck I–IV injuries, whereas PCL reconstruction was associated with stiffness. Engebretsen et al. [13] described a trend toward medial sided injuries. This could not be confirmed when dividing patients into subgroups based on Schenck’s Classification (Table 4). In accordance, Richter [70] and Jokela [41] et al. found no increased rates in Schenck IIIM injuries.

**Table 4:** Cases of AF grouped by Schenck Classification

Schenck grade	Total number of patients (author)	Number of cases with AF (rate in %)
II	31 (Harner)	4 (12.9)
IIIM	33 (Richter, Jokela)	3 (9.1)
IIIL	23 (Okoshi, Ibrahim)	5 (21.7)
IV	9 (Huax)	3 (33.3)

### Timing of surgery

Injuries treated after an initial non-operative rehabilitation (> 3 weeks) tended to develop lower rates of AF with 9.5% compared to injuries treated surgically in the acute phase with 12.7% (Table 3). However, the highest prevalence of AF was found after staged treatment algorithms with 18.0%. Harner and Patterson et al. [22, 65] provided evidence that primarily acutely treated patients required MUA.

### Surgical technique

The distinction between repair (14.2%) and reconstruction (11.0%) of the torn cruciate ligaments showed a slight trend toward lower rates in the reconstruction group (Table 3).

### Rehabilitation

There was a trend showing an increased rate of AF (14.0% vs. 11.6%) in studies compelling a more strict rehabilitation program with postoperative ROM allowed ≤ 90° within the first three postoperative weeks (Table 3).

### Treatment of AF

The time from index surgery to MUA or LOA in the included studies averaged at 14.3 ± 8.8 (range 5–30) and 27.7 ± 12.8 (range 4–52) weeks, respectively. AF was mostly treated surgically by LOA (78.5%) and less often with MUA (21.5%). The total ROM before MUA and LOA was 51.7° ± 23.5 (range 30–90) and 80.2° ± 17.0 (range 60–113), resulting in total ROM gain of 65.0° ± 19.7 (range 22–65) and 48.0° ± 10.6 (range 33–60), respectively. There was no report of any complications after MUA/LOA (Table 5).

**Table 5:** Summary of collected data concerning the prevalence

Author	AF in % (*n*)	Time to MUA in weeks	Time to LOA in weeks	ROM before MUA	ROM before LOA	ROM gain after MUA	ROM gain after LOA
Shapiro et al	57.1 (4)	6.5	26.0	30°	60°	100°	59°
Wascher et al	15.4 (2)	–	36.9	–	95°	–	35°
Yeh et al	13.0 (3)	–	13.0	–	n/a	–	n/a
Ohkoshi et al	12.5 (1)	5.0	–	90°	–	40°	n/a
Harner et al	12.9 (4)	9.7	30.0	n/a	n/a	55	n/a
Talbot et al	10.0 (2)	–	4.0	–	n/a	–	n/a
Bin et al	21.4 (3)	–	n/a	–	113°	–	n/a
Ibrahim et al	15.0 (3)	–	n/a	–	n/a	–	n/a
Lo et al	9.1 (1)	–	21.7	–	75°	–	33°
Engebretsen et al	6.0 (5)	n/a	n/a	n/a	n/a	n/a	n/a
Ranger et al	19.7 (14)	–	n/a	–	n/a	–	n/a
Ibrahim et al	20.0 (4)	–	n/a	–	n/a	–	n/a
Piontek et al	9.1 (1)	–	52.1	–	n/a	–	n/a
Werner et al	15.4 (10)	–	n/a	–	n/a	–	n/a
Heitmann et al.2014	12.5 (1)	–	n/a	–	n/a	–	n/a
Richter et al	12.5 (1)	n/a	–	n/a	–	n/a	–
Kohl et al	5.7 (2)	–	n/a	–	73°	n/a	53°
Angelini et al	7.1 (1)	30.4	n/a	35°	–	65°	–
Khakha et al	2.8 (1)	n/a	-	n/a	–	n/a	–
Huax et al	18.8 (3)	20.0	39.0	n/a	n/a/	n/a	n/a
Sundararajan et al	4.5 (2)	–	n/a	–	65°	-	60°
Heitmann et al.2019	11.6 (8)	n/a	n/a	n/a	n/a	n/a	n/a
Jokela et al	8.0 (2)	–	–	–	–	–	–
Rosteius et al	18.5 (5)	–	26.9	–	n/a	–	n/a
Goyal et al	11.1 (3)	n/a	–	n/a	–	n/a	-
Mean Total ± SD (range)	12.1 (2.8–57.1)	14.3 ± 8.8 (5–30)	27.7 ± 12.8 (4–52)	51.7° ± 23.5 (30–90)	80.2° ± 17.0 (60–113)	65.0° ± 19.7 (40–100)	48.0° ± 10.6 (33–60)

AF. Additionally, the distribution of MUA and LOA, time from index surgery to MUA/LOA, total ROM before and total ROM gain after MUA/LOA (at final follow-up) are shown

*“n/a” indicates no data available, while "-” indicates no reported cases in the selected study;*

### Reporting quality and assessment of bias

Among the included studies, 15 were retrospective case series, five retrospective cohort studies and five prospective studies (Table 2).

The most common reasons for deduction in the Coleman score were study size < 60 patients (22/25 studies), absence of randomized controlled studies (25/25), retrospective collection of data (21/25 studies) and completion of assessment by subjects with minimal investigator assistance (24/25). The overall mean Coleman score of all included studies was 56.2 ± 12.5 points. Scores ranged from 84 to 31 points, showing a trend for better scores in more recent studies. Overall three studies were rated as good (12.0%), 16 as fair (64.0%) and 6 as poor (24.0%).

The interrater agreement was kappa = 0.922 (*p* < 0.001), implying a nearly perfect agreement between the two independent assessors (H.F. and L.K.) [47].

## Discussion

Primary findings of this review demonstrate that (1) AF is a poorly defined condition with lack of consistency in the literature. (2) The absolute risk of AF across all studies was 12.1%. (3) Potential risk factors for AF are knee dislocation, higher-grade injuries (Schenck III and IV), acute treatment and ROM limiting rehabilitation programs. (4) Treatment of AF was mostly done by LOA (78.5%). (5) MUA and LOA were performed at an average of 14.3 and 27.7 weeks, respectively, and are safe and easy methods which yield good results. (6) The overall methodological quality of the studies was poor as measured by the Coleman score.

Our results confirm a recent meta-analysis by Kim et al. [45], who showed in an analysis of 21 studies pooled rates of AF to be 11.2%. However, Kim included studies with disruption of at least 2 of the 4 major knee ligaments and provided no further information about risk factors and treatment options.

### Definition

There is a great inconsistency in defining AF in the included studies and literature in general [12, 82]. Ekhtiari et al. [12] came to similar results in a systematic review focusing on AF following ACL reconstruction and endorsed a homogenous definition and treatment guidelines for AF.

The terms “arthrofibrosis,” “stiffness” and “loss of motion” are often confusing, misleading and used synonymously. In the literature, complications concerning loss of motion, regardless of histopathological origin, time after surgery or, treatment responsiveness, are most commonly referred to as stiffness or AF [12, 57]. There are many classifications regarding motion loss [21, 40, 70, 77], but none does consider the multimodality of this condition.

It is worth mentioning that early ROM deficiencies within 3 months postoperatively are another entity, since these are often caused by postoperative swelling, pain or potential ROM restrictions given by the surgeon [61]. They can often be successfully treated by intensive physiotherapy and rarely need interventions like MUA/LOA [61]. Other factors like mechanical blockage (e.g., malposition of drilling tunnels, obstructive endobuttons, heterotopic ossifications) and infection also lead to loss of motion and are important differential diagnosis of AF. Hence, we propose a simply applicable objective clinical definition of AF including (1) motion loss defined by Shelbourne’s original classification [77], (2) occurrence > 3 months postoperatively, (3) absence of mechanical blockage or infection and (4) insufficient improvement of ROM by aggressive physiotherapy.

### Injury pattern

In terms of injury pattern, a trend toward higher rates of AF in Schenck III–IV injuries was detected. This is supported by an epidemiology study, where a doubling of the 6-month incidence of MUA after combined ACL and collateral ligament reconstruction (1.8%), concomitant arthroscopic ACL and PCL reconstruction (4.1%), and combined ACL, PCL and collateral ligament reconstruction (8.0%) was shown [85]. Injuries with three or more ligaments requiring operative intervention and knee dislocation were shown to be associated with stiffness [9, 21], whereas others could not detect a correlation with the knee dislocation grade and postoperative motion loss [32]. Engebretsen et al. [13] suspected medial sided injuries to be associated with higher rates of AF, which is supported by a large retrospective study where mostly Schenck IIIM injuries required LOA [27]. However, this trend could not be supported by our results as well as by a large study of La Prade et al., who included surgically treated patients with at least two major knee ligaments torn [48]. A reason for this inconsistency could be the heterogeneity of the studies, e.g., in terms of surgical technique and rehabilitation; however, lateral injuries may require more complex surgery than medial sided injuries (e.g., reconstruction of the posterolateral corner) and therefore result in higher rates of AF.

A possible explanation for more severe injuries to have higher rates of AF is extensive soft tissue damage resulting in greater inflammatory reaction and longer operation time.

### Surgical technique

Our data suggest a minor difference between reconstruction and repair of torn cruciate ligaments favoring reconstruction, which could be explained by the connection between acute treatment and repair, resulting in higher rates of AF.

Other aspects in surgical treatment like procedure time, technique of reconstruction or repair were unrewarding to investigate since the included studies showed great heterogeneity and provided scarce information.

Interestingly, allografts tolerate long periods of immobilization better than autografts and minimization of autografts taken from the injured knee is described to decrease the risk of AF [54, 73]. Another recently published study showed that prolonged procedure time (> 300 min), PCL reconstruction and shorter time to surgery were particular risk factors [3]. Taken together the impact of the surgical technique remains elusive, but allografts and short surgery time, minimizing the soft tissue damage, might prevent the development of AF.

### Timing of surgery

Many studies and systematic reviews advocate that acute surgery yields overall better results [22, 28, 44, 50, 79] and delayed surgery (after 2–3 weeks) decreases the risk of AF [14, 50], whereas others found that acute surgery was not associated with stiffness [21].

The included studies indicate a trend toward higher rates of AF in acutely treated MLKI. This is in line with a review, showing that the number of patients undergoing MUA or LOA for the treatment of joint stiffness is greater in both the staged and acute treatment groups compared with the chronic treatment group [59]. Similar results were presented with all patients requiring manipulation or surgery for AF, who had surgical repair in the acute phase but higher outcome scores compared with those receiving surgery later [48, 63]. These results are underlined by a study focusing on the complications after MLKI surgeries [64], which showed a significantly decreased risk of AF when time to surgery is delayed.

One possible explanation for lower AF rates in delayed surgery could be related to more extensive preoperative physical therapy, which could ultimately result in better postoperative ROM. Additionally, increased time from injury to surgery may allow for reduced inflammation and swelling in the knee as shown in ACL reconstruction surgery (ACLR) [33].

Hence, a staged procedure was proposed, which might combine the advantages of acute and chronic treatment [78]. Mainly, this means reconstruction/repair of the PCL and peripheral ligaments, while the ACL is reconstructed later or treated conservatively [39]. Surprisingly, we found the highest rates of AF (18.2%) within staged treatment. Noticeably, only two studies in this review [5, 62] conducted a staged treatment, with a small group of patients (*n* = 22), which may lead to relevant bias. Mook et al. [59] showed in a systematic review lower rates of flexion loss in staged treatment, but no difference in AF, apart from this pointing out the importance of distinguishing between simple flexion loss and AF. A review concluded that staged treatment simplified the operative process and operation time and therefore might decrease the rate of AF [39]. However, a study comparing acute and staged treatment could not detect a difference in postoperative ROM between acute and staged treatment [37].

We propose that best results are shown when surgery is performed as soon as the patient general condition allows them to actively participate in postoperative rehabilitation. Solely if ligament surgery is unavoidably delayed and stability is crucial (e.g., vascular injury, compartment syndrome, open major trauma, general condition), the knee should be stabilized within a hinged external fixator or hinged knee brace, thereby accepting potential motion loss [31, 55].

### Rehabilitation

After MLKI surgery, a balance between the risk of recurrent laxity and the risk of AF must be found [20]. Aggressive motion exercise started too early might risk stretching or damaging the healing soft tissue. Accordingly, timing and intensity of postoperative rehabilitation play a crucial role in terms of final outcome [7, 9, 14, 71].

Our data provide evidence that a more aggressive rehabilitation may lead to lower rates of AF while the final outcome is unaffected.

In accordance, a systematic review discovered a difference in flexion loss among patients when compared on the basis of rehabilitation [59]. Within the acute treatment group, flexion loss of 10° was reported in 48.0% of those who were immobilized, compared with 28.0% of those who were allowed early mobilization. Encouragingly early rehabilitation was not associated with increased joint instability in acutely managed patient [59]. A recent review showed consistently improved outcomes after early postoperative physical therapy and range of motion [42]. Hoit et al. [29], however, were unable to demonstrate a difference between early and late knee rehabilitation with regard to knee stiffness, laxity or patient-reported outcomes, but it is worth mentioning they had a very limited study size of 36 participants.

Taken together there is evidence that aggressive postoperative rehabilitation leads to lower rates of AF, without altering patient-reported outcomes. Not to be forgotten treating residual laxity is often more challenging than AF [74].

### Treatment of AF

Paulus et al. [66] proposed that early surgical intervention to treat AF is best. In the literature, AF is described to require surgical treatment in 29.0% of patients [7]. It was shown that the results of LOA procedure yield excellent motion gains and surgery should be performed sooner than six months after primary surgery to achieve best outcome [10]. Time from primary surgery to MUA and LOA in the included studies averaged at 14.3 and 27.7 weeks with LOA being performed in the majority of cases. Similarly in total knee arthroplasty, if MUA has not been performed within 3 months or is unsuccessful, LOA is recommended, because the risk of iatrogenic fracture during MUA increases [4]. It is assumed that similar treatment algorithms and outcomes can be assigned to knee ligament surgery [12]. When comparing MUA and LOA (with or without concurrent MUA), the final ROM is similar [17]. The greater ROM gain shown in MUA compared with LOA in this review might be due to the severely limited ROM in cases with MUA before the intervention. A differentiation between flexion and extension could not be performed due to the poor study situation. However, this is important since a loss of flexion is easier to treat and usually better tolerated [36].

Hughes et al. [32] noticed, in a retrospective case series, time from primary surgery to MUA/LOA was 13.7 weeks, with no distinction between MUA and LOA being made. These results are only comparable to a limited extent, since Hughes et al. included 61% KD I injuries. Furthermore, it was shown that motion loss in the early stages (1 month postoperatively) was treated mostly conservatively but in later stages (3–6 months) all cases were treated with MUA/LOA, thus emphasizing the importance of early and aggressive physical therapy if motion loss is encountered within the first 3 months postoperatively. Overall, MUA and LOA are safe and well established procedures which yield excellent results.

## Limitations

First a limitation of this systematic review is reliance on data from Level III and IV evidence studies; this is due to a general lack of high level of evidence studies related to MLKI. The low methodological quality of the included studies, lack of control groups and of randomization limits the quality of our results. Secondly, there was an essential amount of heterogeneity within the patient characteristics, injury mechanism and surgical technique. Thirdly, the detected differences in AF especially in terms of surgical technique, timing and rehabilitation should be treated with caution, due to the slight differences in small number of cases. Additionally, the reporting of AF and outcomes was often inconsistent and poor, which affected our analysis. It is important to mention that none of the included studies made direct comparisons between groups with regard to AF. AF definition was mostly simplified (requirement of LOA/MUA) not considering the pathologies multimodality and fluent transition between loss of motion and AF. Moreover, many studies did not report about AF and had to be sorted out, resulting in a selection bias. For future investigations, larger prospective studies with higher methodological quality and detailed information about AF are needed.

## Conclusion

AF is a common but poorly defined complication particularly in high-grade MLKI. Early postoperative and intensified physiotherapy is important to reduce the risk of AF. MUA and LOA are very effective treatment options and result in good clinical outcome. Prospective studies with bigger study population are needed to optimize treatment algorithms of further patients after MLKI.
